# Disclosure, reporting and help seeking among child survivors of violence: a cross-country analysis

**DOI:** 10.1186/s12889-020-09069-7

**Published:** 2020-07-02

**Authors:** Audrey Pereira, Amber Peterman, Anastasia Naomi Neijhoft, Robert Buluma, Rocio Aznar Daban, Aminul Islam, Esmie Tamanda Vilili Kainja, Inah Fatoumata Kaloga, They Kheam, Afrooz Kaviani Johnson, M. Catherine Maternowska, Alina Potts, Chivith Rottanak, Chea Samnang, Mary Shawa, Miho Yoshikawa, Tia Palermo

**Affiliations:** 1 International Food Policy Research Institute, 1201 I St., NW, Washington, DC 20005-3915 USA; 2 UNICEF Office of Research—Innocenti, Piazza SS Annunziata 12, 50122 Florence, Italy; 3 Department of Public Policy, Abernethy Hall CB #3435, University of North Carolina at Chapel Hill, Chapel Hill, 27599 USA; 4 UNICEF Mozambique, 1440 Av. do Zimbábwe, Maputo, Mozambique; 5 Kenya National Bureau of Statistics, P.O. Box 30266–00100 GPO, Nairobi, Kenya; 6 UNICEF New York Headquarters, 3 UN Plaza, New York, 10017 USA; 7 UNICEF Kenya, P.O. Box 44145-00100, Nairobi, Kenya; 8 Ministry of Gender, Children, Disability and Social Welfare, Lilongwe 3, Malawi; 9 UNICEF Nepal, UN House, Pulchowk, P.O. Box 1187, Lalitpur, Kathmandu, 44700 Nepal; 10 Demographics Statistics Census and Survey Department, National Institute of Statistics, Ministry of Planning, #386 Preah Monivong Blvd, Boeung Keng Kong 1, Chamkarmorn, Phnom Penh, Cambodia; 11 UNICEF Malawi, Mantino Complex, Area 40/31, Lilongwe 3, Malawi; 12 Global Partnership to End Violence Against Children, Avenue de la Paix 5 - 7, 1202 Geneva, Switzerland; 13 The Global Women’s Institute, 2140 G Street NW, Washington, DC 20052 USA; 14 UNICEF Cambodia, 5th Floor, Exchange Square, Building No. 19&20, St 106, Phnom Penh, Cambodia; 15 Department of Social Work, Royal University of Phnom Penh, Russian Federation Boulevard, Toul Kork, Phnom Penh, Cambodia; 16 Department of Epidemiology and Environmental Health, 270 Farber Hall, University at Buffalo (State University of New York), Buffalo, NY 14214 USA

**Keywords:** Disclosure, Gender-based violence, Help-seeking, Violence against children

## Abstract

**Background:**

Violence against children is a pervasive public health issue, with limited data available across multiple contexts. This study explores the rarely studied prevalence and dynamics around disclosure, reporting and help-seeking behaviours of children who ever experienced physical and/or sexual violence.

**Methods:**

Using nationally-representative Violence Against Children Surveys in six countries: Cambodia, Haiti, Kenya, Malawi, Nigeria and Tanzania, we present descriptive statistics for prevalence of four outcomes among children aged 13–17 years: informal disclosure, knowledge of where to seek formal help, formal disclosure/help seeking and receipt of formal help. We ran country-specific multivariate logistic regressions predicting outcomes on factors at the individual, household and community levels.

**Results:**

The prevalence of help-seeking behaviours ranged from 23 to 54% for informal disclosure, 16 to 28% for knowledge of where to seek formal help, under 1 to 25% for formal disclosure or help seeking, and 1 to 11% for receipt of formal help. Factors consistently correlated with promoting help-seeking behaviours included household number of adult females and absence of biological father, while those correlated with reduced help-seeking behaviours included being male and living in a female-headed household. Primary reasons for not seeking help varied by country, including self-blame, apathy and not needing or wanting services.

**Conclusions:**

Across countries examined, help-seeking and receipt of formal services is low for children experiencing physical and/or sexual violence, with few consistent factors identified which facilitated help-seeking. Further understanding of help seeking, alongside improved data quality and availability will aid prevention responses, including the ability to assist child survivors in a timely manner.

## Background

Children across all ages experience violence in a range of settings, from various perpetrators, including from parents/guardians, peers and intimate partners [1]. Survivors of such abuse can experience physical, psychological and behavioural consequences that persist into adulthood [2–7]. A recent six-country study using the Violence Against Children Surveys (VACS) showed that lifetime prevalence of physical violence among children aged 13 to 17 years ranged from 50 to 84%, while that for sexual violence ranged from 6 to 36% [8]. Further, a systematic review suggests that in developing countries, an excess of 1 billion children under the age of 18 experience emotional, physical or sexual violence annually [9]. Despite these high figures, nearly all research is presented with the caveat that estimates are likely to be a lower-bound of the true prevalence; violence against children (VAC) is underreported, under-acknowledged, and “hidden in plain sight” [1, 10].

The literature on help-seeking behaviours also remains sparse. Studies show children may not disclose violence for many interrelated and contextual reasons, including failure to recognize abuse as a problem or believe they are in need of services, normalization of violence, lack of vocabulary to describe abuse, fear of repercussions either for themselves or the perpetrator, shame, stigma, and self-blame [11–13]. Other barriers to disclosure and help seeking include lack of social support (i.e.*,* not having anyone to turn to for help), lack of access to services (i.e.*,* physical or financial constraints), and perceived helplessness (e.g. distrust of services, or thinking nothing will change) [11, 14, 15]. In contrast, factors promoting disclosure or help seeking include changes in children’s development, the nature (or severity) of abuse, intervention from individuals who notice symptoms of abuse or regression in the child’s behaviour, and the need to protect other children from violence at the hands of the same perpetrator [11]. The importance of such factors likely varies depending on the severity and recurrence of violence, the child’s relationship with the perpetrator, and the environment in which the child lives (including social norms around violence).

UNICEF’s *Hidden in Plain Sight* report explored data from 20 low- and middle-income countries (LMICs) using Demographic and Health Survey data, and shows that a large proportion of adolescent girls aged 15 to 19 years never disclosed or sought help following experiences of physical and/or sexual violence (ranging from approximately 32–69%) [10]. Girls were less likely to come forward if they had experienced sexual violence alone, compared to physical violence alone or physical and sexual violence. A recent longitudinal study from South Africa examined disclosure and help seeking among victims of emotional, physical and sexual abuse, and found that although 99% of children in the study sample knew of available services, only 20% of those who were abused disclosed and accessed help, while 14% actually received help. Girls were more likely to seek help than boys; age, poverty and rural residence were not significantly associated with help seeking [16]. In general, the majority of help-seeking studies focus solely on sexual violence [17, 18]. For example, Sumner and colleagues (2015) estimated that only 2.7 to 34% of women and 0.4 to 6.6% of men who reported experiencing sexual violence prior to the age of 18, received any services, using VACS from seven countries [19]. However, determinants of help-seeking behaviour were not explored, further highlighting a gap in existing literature. In addition to ignoring dynamics around experience of multiple violence typologies and poly-victimization, studies tend to focus exclusively on girls, thus we know comparatively less about dynamics for boys or all children’s exposure to violence [17, 18].

This study adds to the growing actionable evidence on VAC in LMICs. This analysis has three objectives: Adding to the literature and available analysis presented in country-level VAC reports, we first estimate the prevalence of distinct help-seeking behaviours and service provision defined as follows: informal disclosure, knowledge of where to seek formal help, formal disclosure or help seeking, and receipt of formal help, among children aged 13–17 years. Estimates of the magnitude of under-reporting help situate the overall burden of VAC within each country and discern how administrative or facility-based data for these same indicators differ from population-based data reported by children themselves. Second, we examine factors at individual, household, and community levels that facilitate or hinder children from help-seeking behaviours. Correlates of help-seeking behaviours from this multivariate framework, which have not been previously analysed in country-level reports or country-specific analyses, help examine how children who report seeking help, and receive services, differ from those who do not. Finally, for individuals who did not seek help, we provide descriptive information regarding the self-reported reasons for not doing so, thus facilitating the development of solutions for policy or programming to overcome them. Taken together, the results can assist programmers and policy makers in preventing VAC and targeting barriers to increase service delivery for survivors, particularly in understanding the role of access, economics, and violence-related social norms.

## Methods

### Context

The prevalence of violence against children in our six countries of interest (Cambodia, Haiti, Kenya, Malawi, Nigeria and Tanzania) remains unacceptably high. Among children aged 13–17 years, the lifetime experience of emotional violence ranged from 20% in Nigeria to 42% in Haiti, among girls, and from 27% in Cambodia and Kenya to 36% in Malawi among boys. The prevalence of physical violence was much higher among both girls and boys: ranging from 60% (Nigeria) to 82% (Tanzania) among girls, and from 58% (Cambodia) to 84% (Malawi) among boys. Approximately 6% (Cambodia) to 36% (Malawi) of girls, and 6% (Cambodia) to 25% (Haiti) of boys reported ever experiencing sexual violence in their lives. In addition, 11% (Kenya) to 53% (Haiti) of girls and 10% (Nigeria) to 49% (Haiti) of boys reported ever experiencing more than one form of violence [8].

### Data

Data comes from cross-sectional and nationally representative VACS in six countries: Cambodia (2013), Haiti (2012), Kenya (2010), Malawi (2013), Nigeria (2014) and Tanzania (2009). VACS are nationally representative surveys of children and young adults designed to measure the prevalence and circumstances surrounding VAC. While there is some variation in questions asked across countries, staff from the Centers for Disease Control and Prevention, UNICEF, the Together for Girls Secretariat and external consultants developed a standardized core questionnaire for maximum comparability across countries [20]. Sample sizes of children 13–17 who experienced any physical and/or sexual violence, and thus included in our analysis were as follows: Tanzania (*n* = 219, where questions regarding help seeking were only asked for sexual violence), Cambodia (*n* = 586), Kenya (*n* = 1005), Haiti (*n* = 1038), Nigeria (*n* = 1185). The VACS also collect information on youth aged 18–24 years, however because they ask only about retrospective violence experienced before age 18, this sample is not appropriate for conducting determinant analysis as there is a mismatch between current characteristics and past violence and help-seeking experiences. All questionnaires follow the core standardized VAC questionnaires and were further adapted and tested locally under the guidance of a national technical advisory group to ensure cultural relevance and accuracy of questions. The six countries were chosen based on data availability for secondary analysis and government interest to participate in this cross-country study. The VACS are conducted with national statistics institutes or national academic partners, and follow strict ethical protocol related to participant safety, confidentiality and response plans. All countries conducted national surveys using multi-stage cluster sample survey designs, with sampling frames from the most recent, or most recent and updated, national census. Pilot tests were conducted in all countries to test the questionnaire and referral processes prior to data collection. Interviewers were trained at length on the background and purpose of the study, procedures for and importance of maintaining privacy during the interview and confidentiality, sensitivity toward study subjects, referral services and procedures, human subjects research protection, and other topics. Consent for interviews was first obtained from parents or primary caregivers and then informed assent was obtained from participants. Surveys were administered in one or more local languages. Each country implemented in person same-sex surveys (face-to-face interviews where enumerators are matched to children of the same sex) which ask a range of questions related to violence and background characteristics. Individual response rates ranged from 84% in Malawi to 96% in Nigeria for females and from 83% in Malawi to 97% in Nigeria for males. More information on questionnaire adaptation, enumerator training, and data collection procedures is available in each country report [21–26]. Appendix A details information on sampling, survey implementation, ethical assurances, cluster and split-sample design, and prevalence of lifetime violence by type and sex for each country. Our analysis is limited to respondents who reported ever experiencing physical and/or sexual violence (as per country-specific definitions available in Appendix B). Due to sample size limitations, it was not possible to analyse determinants of help-seeking behaviours following experiences of physical and sexual violence separately. However, we provide means of help-seeking behaviours by type of violence in Appendix H-I.

### Key indicators

We conducted analyses for each help-seeking behavioural outcome (as defined in Appendix B). Specifically, we examine four self-reported help-seeking outcomes, with slight variations depending on data availability, by country: 1) informal disclosure (e.g., to family, friends, neighbours, community/religious leaders), 2) knowledge of where to seek formal help (e.g., legal, health or social services), 3) formal disclosure or help seeking (e.g. hospital/clinic, police station, social worker), and 4) receipt of formal help. Questions regarding help-seeking behaviours typically followed physical and sexual violence, however in some cases, questions were asked only following one type of violence. General definitions of outcomes, covariates and reasons for not seeking help are provided in Table 1. Specific question wording of outcomes by violence type is provided by country in Appendix C-D. Because of differences in wording in violence and health seeking definitions and hence indicators, as well as samples between countries, results between countries are not strictly comparable. For example, physical peer violence was not asked about in Cambodia, Haiti or Kenya, while physical intimate partner violence (IPV) was not asked about in Haiti. These are only some of the variations further detailed in Appendix B.

**Table 1:** Definitions of Outcomes, Covariates and Reasons for not Seeking Help

**Outcomes**	
Informal disclosure	Have you ever told anyone about these experiences [of violence]? Responses: e.g. family, relatives, partners, friends, neighbours.
Knowledge of where to seek formal help	Do you know a hospital or clinic, police station, social worker, teacher child protection network, NGO or FBO to go to for help? Responses: [1 = Yes; 0 = No]
Formal disclosure or help seeking	Have you ever told anyone about these experiences [of violence]? Responses: e.g. hospital/clinic, police station, helpline, social welfare or legal office. Did you try to seek professional help for any of these incidents [of violence]? Responses: [1 = Yes; 0 = No]
Receipt of formal help	Were you successful in receiving any professional help for any of theseincidents [of violence]? Responses: [1 = Yes; 0 =No]
**Covariates**	
Age	Years (numerical)
Educational status	Current school enrolment [1 = Yes; 0 = No]
Parental absence	Biological mother deceased or not living at home [1 = Yes; 0 = No] Biological father deceased or not living at home [1 = Yes; 0 = No]
Household composition	Number of children under age 18 years in household Number of adult females aged 18 and over in household Number of adult males aged 18 and over in household
Female-headed household	[1 = Yes; 0 = No]
Household wealth quintiles	Wealth quintiles are from indices created through factor analysis of household assets and dwelling characteristics, similar to standard methodology from the Demographic and Health Surveys for the analysis sample.
Urban residence	[1 = Urban; 0 = Rural]
Camp	Haiti only; Internally displaced person living in caps/tent settlements resulting from the 2010 earthquake [1 = Yes; 0 = No]
Zanzibar	Tanzania only; [1 = Zanzibar; 0 = Mainland]
**Reasons for not seeking help**	
Lack of awareness	1) Did not know where to go
Lack of access	1) Too far to services
Afraid of repercussions	1) Afraid of causing more violence or getting into trouble; 2) Did not want perpetrator to get into trouble; 3) Was or felt threatened/threatened by perpetrator
Shame and stigma	1) Afraid of being mocked; 2) Embarrassed for self or family; 3) Felt ashamed
Self-blame	1) Felt it was my fault
Financial constraints	1) Could not afford services; 2) Could not afford transport
Lack of social support	1) Afraid of being abandoned; 2) Dependent on perpetrator; 3) No one to help me
Apathy	1) Did not think it was a problem
Perceived helplessness	1) Felt it was useless
Did not need or want services	1) Did not need or want services
Other	1) Other reasons for not seeking help

Abbreviations: *FBO* faith-based organization; *NGO* non-governmental organization;

Notes: ^a^Indicators comes from a nationally representative sample of children aged 13–17 years from Violence Against Children Surveys. Male and female respondents are asked identical questions

^b^Specific questions for outcomes, by violence type and country, are available in Appendix C-D

^c^Questions on reasons for not seeking help were not asked in Tanzania

Reasons for not seeking help were categorized as follows: 1) lack of awareness (did not know where to go), 2) lack of access (too far to services), 3) afraid of repercussions (causing more violence or getting into trouble; did not want perpetrator to get into trouble; threatened by perpetrator; was or felt threatened), 4) shame and stigma (afraid of being mocked; embarrassed for self or family; felt ashamed), 5) self-blame (felt it was my fault), 6) financial constraints (could not afford services; could not afford transport),7) lack of social support (afraid of being abandoned; dependent on perpetrator; no one to help me), 8) apathy (did not think it was a problem), 9) perceived helplessness (felt it was useless), 10) did not need or want services, and 11) other reasons. Reasons for not seeking help were not asked in Tanzania.

We explore the following determinants of help-seeking: age (years), current school enrolment, parental absence (separate variables coded as one if mother or father were absent), household composition in terms of number of resident children, adult females and adult males, female-headed household, household socio-economic status using wealth quintiles (indices created through factor analysis of household assets and dwelling characteristics), urbanicity. We also controlled for current residence in a camp in Haiti, and living in Zanzibar in Tanzania only. Determinants were chosen based on review of the literature and availability of standardized structural factors at different levels across countries in the VACS data. Descriptive statistics for all control variables by country are reported in Appendix E, F, G.

### Statistical analysis

We conducted country-specific multivariate regression (logistic models) and report odds ratios (ORs) with 95% confidence intervals (CIs). We accounted for complex survey design by adjusting standard errors for the clustered sample design per the VACS survey guidelines. The guidelines recommend accounting for primary sampling units in all countries, and further accounting for regional stratification of the sample in Haiti and Malawi, and in Tanzania by mainland versus Zanzibar. In addition, we performed weighted descriptive analyses accounting for complex survey design for prevalence levels on help-seeking and on why children did not seek help. Our analysis sample only includes individuals who reported experiencing physical and/or sexual violence and had no missing values for all help-seeking outcomes. Wealth quintiles were imputed for missing values using factor analysis for the analysis sample in Kenya only (< 1%). We used Stata version 14 for all analyses.

## Results

### Prevalence of help-seeking behaviours

Table 2 provides the prevalence of our four outcomes by country. Informal disclosure ranged from 23% (Cambodia) to 54% (Malawi) and knowledge of where to seek formal help ranged from 16% (Kenya) to 28% (Malawi). Cambodia had the lowest mean for formal disclosure or help seeking at less than 1%, while the highest mean was 25% (Tanzania). Finally, across countries, receipt of formal help was low, ranging from 1% (Nigeria) to 11% (Tanzania) (question not asked in Cambodia and Haiti). The prevalence of the four outcomes by country, disaggregated by type of violence are provided in Appendix H-I.

**Table 2:** Descriptive Statistics for Help-Seeking Behaviors Among Children Aged 13–17 Years Experiencing Physical and/or Sexual Violence by Country

Country		Informal: Disclosure	Formal: Knowledge of where to seek help	Formal: Disclosure or help seeking	Formal: Received help
Cambodia	Prevalence	23.05	na	0.11	na
	95% CI	19.28, 26.82		−0.1, 0.32	
	N	586		586	
Haiti	Prevalence	42.40	na	8.60	na
	95% CI	35.37, 49.42		6.24, 10.97	
	N	385		1038	
Kenya	Prevalence	31.67	16.26	4.74	2.06
	95% CI	21.70, 41.64	13.02, 19.50	2.24, 7.25	0.96, 3.16
	N	191	1005	1005	1005
Malawi	Prevalence	54.13	27.92	12.34	7.49
	95% CI	48.40, 59.87	23.00, 32.83	8.04, 16.65	3.47, 11.51
	N	864	864	864	864
Nigeria	Prevalence	40.55	18.09	4.12	1.46
	95% CI	36.69, 44.41	14.82, 21.35	2.82, 5.42	0.63, 2.29
	N	1185	1185	1185	1185
Tanzania	Prevalence	42.04	na	25.30	11.22
	95% CI	30.45, 53.63		16.31, 34.28	3.99, 18.45
	N	219		219	219

Notes: ^a^Estimates are prevalences with corresponding 95% Confidence Intervals

^b^Data comes from a nationally representative sample of children and youth aged 13–24 years from Violence Against Children Surveys. Samples from male and female respondents are combined due to low overall help seeking rates within subsamples

^c^Cambodia did not ask questions on knowledge of where to seek formal help or on receiving formal help

^d^In Haiti, informal disclosure was asked for experience of sexual violence only. Due to the wording of questions, we were unable to parse out formal disclosure from receiving help for experience of physical violence. Haiti did not ask questions on knowledge of where to seek help

^e^In Kenya, disclosure, both formal and informal, was asked for experience of sexual violence only

^f^In Tanzania, help seeking questions were asked for experience of sexual violence only. Questions on knowledge of where to seek formal help were not asked. Due to errors in survey administration, and in order to remain aligned with the Tanzania VACS report, our sample excludes those who experienced sexual violence prior to the age of 18 and were not asked about disclosure and help seeking behaviours for their experience of violence

### Determinants of help-seeking behaviours

Tables 3-4 show results from logistic regressions for the four outcomes by country. Due to the volume of results, we discuss patterns only where at least three countries show significant relationships of any given determinant across the four outcomes examined. Males were less likely than females to informally disclose in Haiti, Kenya and Tanzania [OR range: 0.21 (CI: 0.08, 0.51) in Kenya to 0.38 (CI: 0.22, 0.67) in Haiti], and in Tanzania, to formally disclose or seek help [OR: 0.24 (CI: 0.06, 0.97)]. However, males were more likely than females to know where to seek formal help in Malawi [OR: 1.67 (CI: 1.03, 2.71)] and Nigeria [OR: 2.29 (CI: 1.42, 3.70)]. There were no differences between males and females in rates of receiving formal help in any country. Increasing age (in years) was positively associated with informal disclosure only in Nigeria [OR range: 1.13 (CI: 1.02, 1.25)] and knowledge of where to seek formal help in Malawi [OR: 1.39 (CI: 1.15, 1.68)].

**Table 3:** Logistic Models Predicting Help-Seeking Behaviors Among Children Aged 13–17 Years Experiencing Physical and/or Sexual Violence in Cambodia, Haiti, and Kenya

	Cambodia	Haiti		Kenya			
	(1)	(1)	(2)	(1)	(2)	(3)	(4)
	Informal: Disclosure	Informal: Disclosure	Formal: Disclosure or received help	Informal: Disclosure (sexual violence only)	Formal: Knowledge of where to seek help	Formal: Disclosure or help seeking	Formal: Received help
Male	0.76	0.38**	0.57	0.21**	1.10	0.66	0.88
	(0.48, 1.20)	**(0.22, 0.67)**	(0.31, 1.04)	**(0.08, 0.51)**	(0.71, 1.72)	(0.26, 1.67)	(0.26, 3.05)
Age in years	1.02	0.95	1.14	0.85	1.09	1.18	0.85
	(0.85, 1.22)	(0.76, 1.18)	(0.92, 1.41)	(0.62, 1.17)	(0.92, 1.30)	(0.76, 1.81)	(0.53, 1.36)
Currently enrolled in school	0.69	1.72	0.39**	1.18	1.95*	2.38	0.94
	(0.40, 1.20)	(0.84, 3.52)	**(0.22, 0.72)**	(0.40, 3.55)	**(1.00, 3.78)**	(0.56, 10.20)	(0.24, 3.69)
Not living with biological mother	1.16	0.92	0.89	3.16	1.33	2.63	1.47
	(0.54, 2.49)	(0.48, 1.77)	(0.48, 1.63)	(0.60, 16.56)	(0.72, 2.45)	(0.82, 8.48)	(0.55, 3.95)
Not living with biological father	1.24	1.71	0.93	0.45	0.90	1.44	1.70
	(0.50, 3.07)	(0.95, 3.07)	(0.53, 1.64)	(0.10, 2.12)	(0.49, 1.65)	(0.61, 3.37)	(0.66, 4.38)
Number of children 0–17 years in household	0.88	0.95	0.99	1.27	1.10	1.18	1.04
	(0.74, 1.06)	(0.78, 1.15)	(0.86, 1.13)	(0.94, 1.72)	(0.94, 1.27)	(0.96, 1.45)	(0.75, 1.42)
Number of males 18+ in household	0.88	0.96	0.94	‡	‡	‡	‡
	(0.63, 1.21)	(0.75, 1.24)	(0.72, 1.24)	‡	‡	‡	‡
Number of females 18+ in household	0.73	0.94	1.11	‡	‡	‡	‡
	(0.52, 1.02)	(0.69, 1.28)	(0.80, 1.54)	‡	‡	‡	‡
Number of adults 18+ in household	†	†	†	1.08	1.07	1.10	1.36
	†	†	†	(0.83, 1.39)	(0.92, 1.23)	(0.84, 1.45)	(0.97, 1.92)
Female-headed household	0.58	0.60	0.71	1.56	1.22	0.56	0.14**
	(0.24, 1.37)	(0.32, 1.11)	(0.38, 1.32)	(0.55, 4.41)	(0.63, 2.35)	(0.19, 1.69)	**(0.04, 0.55)**
Poorer wealth quintile *(base = poorest)*	2.19*	0.51	0.97	0.68	0.92	0.23*	0.58
	**(1.00, 4.81)**	(0.21, 1.24)	(0.44, 2.15)	(0.21, 2.23)	(0.46, 1.88)	**(0.06, 0.80)**	(0.12, 2.75)
Middle wealth quintile	1.50	0.53	0.75	0.19**	1.17	0.31	0.05**
	(0.66, 3.40)	(0.23, 1.23)	(0.32, 1.77)	**(0.06, 0.60)**	(0.54, 2.50)	(0.07, 1.27)	**(0.01, 0.36)**
Richer wealth quintile	1.35	0.80	0.51	0.48	1.06	0.44	0.73
	(0.66, 2.73)	(0.31, 2.04)	(0.19, 1.34)	(0.16, 1.49)	(0.51, 2.17)	(0.15, 1.31)	(0.22, 2.41)
Richest wealth quintile	2.39	0.79	0.83	0.67	1.99	0.52	0.81
	(0.96, 5.97)	(0.29, 2.13)	(0.30, 2.26)	(0.19, 2.36)	(0.91, 4.34)	(0.18, 1.46)	(0.23, 2.87)
Urban	1.17	0.69	1.15	1.02	0.88	0.74	1.04
	(0.60, 2.26)	(0.32, 1.49)	(0.51, 2.63)	(0.31, 3.36)	(0.46, 1.68)	(0.25, 2.15)	(0.24, 4.51)
Camp^g^	§	2.12	2.26	§	§	§	§
	§	(0.97, 4.63)	(0.77, 6.62)	§	§	§	§
Observations	586	385	1040	191	1005	1005	1005

Notes: ^a^Estimates are odds ratios, from weighted logistic regression models, with 95% Confidence Intervals in e-form in parentheses; ** *P* < 0.01, * *P* < 0.05

^b^Data comes from a nationally representative sample of children aged 13–17 years from Violence Against Children Surveys

^c^Sample only includes observations with no missing values for all outcomes

^d^The number of adults and children in household refer to the number of adults and children in the same sleeping areas as the respondent

^e^Wealth quintiles are from indices created through factor analysis of household assets and dwelling characteristics, similar to standard methodology from the Demographic and Health Surveys and are age-group specific

^f^In Haiti, informal disclosure was asked for experience of sexual violence only. Due to the wording of questions, we were unable to parse out formal disclosure from receiving help for experience of physical violence

^g^Camp variable refers to internally displaced persons living in camps/tent settlements resulting from the 2010 earthquake. This variable is included only in the Haiti analysis

†Included as number of male adults and number of female adults for Cambodia and Haiti

‡The sex disaggregated number of adults is not available in Kenya

§Camp variable is relevant only for Haiti

**Table 4:** Logistic Models Predicting Help-Seeking Behaviors Among Children Aged 13–17 Years Experiencing Physical and/or Sexual Violence in Malawi, Nigeria, and Tanzania

	Malawi				Nigeria				Tanzania		
	(1)	(2)	(3)	(4)	(1)	(2)	(3)	(4)	(1)	(2)	(3)
Covariates	Informal: Disclosure	Formal: Knowledge of where to seek help	Formal: Disclosure or help seeking	Formal: Received help	Informal: Disclosure	Formal: Knowledge of where to seek help	Formal: Disclosure or help seeking	Formal: Received help	Informal: Disclosure	Formal: Disclosure or help seeking	Formal: Received help
Male	0.62	1.67*	0.73	0.72	0.94	2.29**	1.10	1.10	0.29*	0.24*	0.10
	(0.39, 1.01)	**(1.03, 2.71)**	(0.36, 1.49)	(0.29, 1.75)	(0.68, 1.29)	**(1.42, 3.70)**	(0.52, 2.33)	(0.34, 3.61)	**(0.10, 0.82)**	**(0.06, 0.97)**	(0.01, 1.00)
Age in years	1.07	1.39**	1.29	1.45	1.13*	1.12	1.00	0.97	1.38	1.09	1.90
	(0.92, 1.24)	**(1.15, 1.68)**	(0.96, 1.72)	(0.97, 2.16)	**(1.02, 1.25)**	(0.99, 1.27)	(0.79, 1.28)	(0.68, 1.37)	(0.98, 1.94)	(0.73, 1.63)	(0.96, 3.76)
Currently enrolled in school	0.71	1.47	1.26	2.06	1.82**	1.08	1.22	0.75	1.04	1.50	6.32**
	(0.38, 1.34)	(0.74, 2.90)	(0.50, 3.15)	(0.62, 6.83)	**(1.23, 2.69)**	(0.71, 1.63)	(0.50, 2.96)	(0.15, 3.84)	(0.40, 2.70)	(0.57, 3.91)	**(1.98, 20.21)**
Not living with biological mother	1.00	0.96	0.62	0.92	0.70	0.68	1.32	1.34	1.46	0.18**	3.74
	(0.61, 1.65)	(0.51, 1.79)	(0.25, 1.54)	(0.26, 3.31)	(0.44, 1.12)	(0.38, 1.24)	(0.47, 3.72)	(0.24, 7.41)	(0.49, 4.34)	**(0.05, 0.62)**	(0.75, 18.60)
Not living with biological father	1.04	1.04	1.28	0.87	1.36	1.87*	1.10	2.01	1.86	8.10**	0.48
	(0.63, 1.71)	(0.58, 1.86)	(0.51, 3.24)	(0.34, 2.24)	(0.89, 2.07)	**(1.01, 3.44)**	(0.29, 4.22)	(0.20, 20.23)	(0.63, 5.48)	**(2.11, 31.11)**	(0.10, 2.43)
Number of children 0–17 years in household	1.12	1.01	0.83	0.90	0.98	0.98	0.99	0.94	0.95	0.79	0.55*
	(0.99, 1.28)	(0.86, 1.18)	(0.65, 1.06)	(0.69, 1.17)	(0.92, 1.05)	(0.91, 1.07)	(0.84, 1.15)	(0.66, 1.34)	(0.72, 1.25)	(0.59, 1.05)	**(0.34, 0.91)**
Number of males 18+ in household	0.97	0.89	0.78	0.72	1.08	0.93	1.05	1.20	0.84	1.03	1.03
	(0.76, 1.23)	(0.63, 1.25)	(0.44, 1.39)	(0.31, 1.68)	(0.93, 1.25)	(0.78, 1.09)	(0.81, 1.35)	(0.89, 1.63)	(0.50, 1.41)	(0.66, 1.62)	(0.51, 2.12)
Number of females 18+ in household	1.18	1.03	1.43*	1.11	0.96	1.28**	1.04	1.14	1.67*	1.38	1.11
	(0.92, 1.51)	(0.75, 1.39)	**(1.05, 1.95)**	(0.70, 1.76)	(0.83, 1.12)	**(1.08, 1.52)**	(0.81, 1.32)	(0.83, 1.57)	**(1.01, 2.77)**	(0.93, 2.04)	(0.63, 1.94)
Female-headed household	0.84	1.00	0.94	1.29	0.98	0.62	1.42	0.95	1.02	0.23*	0.31
	(0.56, 1.27)	(0.63, 1.60)	(0.38, 2.29)	(0.39, 4.24)	(0.62, 1.54)	(0.35, 1.11)	(0.40, 5.02)	(0.05, 16.84)	(0.35, 2.97)	**(0.05, 0.95)**	(0.05, 1.79)
Poorer wealth quintile *(base = poorest)*	0.69	0.87	2.15	1.55	1.26	1.21	1.86	2.30	1.83	1.80	1.43
	(0.38, 1.28)	(0.47, 1.59)	(0.81, 5.69)	(0.51, 4.66)	(0.76, 2.08)	(0.65, 2.24)	(0.52, 6.68)	(0.33, 16.13)	(0.52, 6.39)	(0.36, 9.13)	(0.16, 12.69)
Middle wealth quintile	1.48	1.27	3.53*	1.82	1.20	1.39	2.62	0.99	4.72**	0.74	1.44
	(0.84, 2.61)	(0.64, 2.49)	**(1.28, 9.75)**	(0.61, 5.45)	(0.74, 1.96)	(0.80, 2.42)	(0.86, 8.02)	(0.12, 8.32)	**(1.46, 15.24)**	(0.12, 4.37)	(0.15, 13.53)
Richer wealth quintile	1.12	2.08	4.14*	3.76*	1.14	1.23	1.30	1.04	4.18*	7.12*	10.38*
	(0.62, 2.05)	(0.91, 4.75)	**(1.31, 13.02)**	**(1.04, 13.67)**	(0.69, 1.89)	(0.68, 2.21)	(0.36, 4.73)	(0.15, 7.19)	**(1.01, 17.35)**	**(1.33, 38.17)**	**(1.05, 102.75)**
Richest wealth quintile	0.37*	1.42	4.10*	3.73	1.32	1.03	1.80	3.86	0.93	1.82	1.26
	**(0.16, 0.86)**	(0.59, 3.41)	**(1.21, 13.84)**	(0.89, 15.64)	(0.77, 2.27)	(0.52, 2.06)	(0.49, 6.60)	(0.51, 29.00)	(0.24, 3.56)	(0.38, 8.71)	(0.19, 8.10)
Urban	2.09*	0.73	0.27*	0.24*	1.09	0.89	0.83	0.68	†	†	†
	**(1.12, 3.93)**	(0.35, 1.50)	**(0.09, 0.80)**	**(0.06, 0.96)**	(0.78, 1.54)	(0.53, 1.49)	(0.38, 1.82)	(0.18, 2.57)	†	†	†
Zanzibar^f^	‡	‡	‡	‡	‡	‡	‡	‡	1.66	0.75	2.44
	‡	‡	‡	‡	‡	‡	‡	‡	(0.55, 5.07)	(0.14, 3.93)	(0.35, 16.96)
Observations	864	864	864	864	1185	1185	1185	1185	217	217	217

Notes: ^a^Estimates are odds ratios, from weighted logistic regression models, with 95% Confidence Intervals in e-form in parentheses; ** *P* < 0.01, * *P* < 0.05

^b^Data comes from a nationally representative sample of children aged 13–17 years from Violence Against Children Surveys

^c^In Tanzania, help seeking questions were asked for experience of sexual violence only. Questions on knowledge of where to seek formal help were not asked

^d^Sample only includes observations with no missing values for all outcomes

^e^Wealth quintiles are from indices created through factor analysis of household assets and dwelling characteristics, similar to standard methodology from the Demographic and Health Surveys and are age-group specific

^f^Zanzibar indicates residence on Zanzibar island, and not mainland Tanzania

†Urban disaggregation is not available for Tanzania

‡Disaggregation for Zanzibar is relevant only for Tanzania

Children enrolled in school were more likely to informally disclose in Nigeria [OR: 1.82 (CI: 1.23, 2.69)], more likely to know where to seek formal help in Kenya [OR: 1.95 (CI: 1.00, 3.78)], and more likely to receive formal help in Tanzania [OR: 6.32 (CI: 1.98, 20.21)], compared to children not enrolled in school. However, in Haiti, those enrolled in school were less likely to disclose or receive formal help than their out-of-school counterparts [OR: 0.39 (CI: 0.22, 0.72)].

In terms of household composition, living without a biological father was positively associated with knowledge of where to seek formal help in Nigeria [OR: 1.87 (CI: 1.01, 3.44)], and formal disclosure and help seeking in Tanzania [OR: 8.10 (CI: 2.11, 31.11)]. Increasing number of adult females in the household was positively associated with increased informal disclosure in Tanzania [OR: 1.67 (CI: 1.01, 2.77)], knowledge of where to seek formal help in Nigeria [OR: 1.28 (CI: 1.08, 1.52)], and formal disclosure or help seeking in Malawi [OR: 1.43 (CI: 1.05, 1.95)]. Living in a female-headed household was negatively associated with formal disclosure or help seeking in Tanzania [OR: 0.23 (CI: 0.05, 0.95)] and with receiving formal help in Kenya [OR: 0.14 (CI: 0.04, 0.55)]. Higher household wealth quintile was at times significantly associated with more favourable help-seeking outcomes, in particular in Nigeria and Tanzania, however this association was not consistent across outcomes and countries.

In Malawi, living in a household in an urban area was positively associated with informal disclosure [OR: 2.09 (CI: 1.12, 3.93], but negatively associated with formal disclosure or help seeking [OR: 0.27 (CI: 0.09, 0.80)] and receiving formal help [OR: 0.24 (CI: 0.06, 0.96)] and not significantly associated with any other outcome. Living in a camp in Haiti, or on Zanzibar instead of mainland Tanzania, were also not significantly associated with any help-seeking behaviours.

### Reasons for not seeking help

Main reasons for not seeking formal help for all countries except Tanzania, where these questions were not asked, are provided in Appendix J-K. In Cambodia, the main reason for not seeking help for physical violence was self-blame (56%), while that for sexual violence was apathy (55%); the second most common reason was shame and stigma for physical violence (12%) and not needing or wanting services for sexual violence (15%). In Kenya, Malawi and Nigeria, the most common reason given for those who experienced physical violence (35–39%) or sexual violence (39–50%) was apathy. The second most common reason for not seeking help for physical violence was being afraid of repercussions (27%) and for sexual violence was shame and stigma (19%) in Kenya. In Malawi, respondents stated other (18–24%) reasons as the second most common reason for not seeking help for both types of violence, while not needing or wanting services (22–24%) was the second most common reason in Nigeria. In Haiti, the most common reason for not seeking help, across both types of violence and different sources of help (counselling, health, law, police), was not needing or wanting services (22–31%), followed by perceived helplessness (16–25%).

## Discussion

Using nationally-representative data from six countries in three regions, this is the first study to systematically examine patterns of disclosure, reporting, and help-seeking among both male and female children experiencing various types of violence. Our results show that overall, prevalence of help-seeking behaviours following experiences of physical and/or sexual violence varies by context, ranging from 23 to 54% for informal disclosure, 16 to 28% for knowledge of where to seek formal help, less than 1 to 25% for formal disclosure or actual help seeking, and 1 to 11% for receipt of formal help. In all countries, levels of informal disclosure are orders of magnitude higher than formal disclosure, signalling that the first point of contact for help-seeking are people children know, including family and friends. Based on the statistics on formal disclosure, using simple computation, results suggest that estimates of physical and/or sexual VAC based on formal reporting mechanisms (e.g.*,* data from health systems or based on police or NGO reporting) may underestimate the total prevalence of VAC, ranging from 4 to 940-fold depending on the country under examination [27]. While this range in multipliers is broad, one concrete message is that the magnitude of underreporting is likely to be large when relying on administrative sources of data. Given the variation in questionnaire designs across countries, these estimates are not strictly comparable across countries. Nevertheless, the trends in disclosure, whereby informal reporting is more common than formal reporting, and the fact that knowledge of where to seek formal support does not surpass one in four survivors in any of the countries studied, underscore that there are major gaps in resources available to childhood violence survivors, and that significant, intersectoral solutions are needed.

With respect to prevalence of help-seeking and disclosure, there are a few key differences between countries, though some minor differences in indicators influence our interpretation of direct comparisons. First, Tanzania has the highest rates of reporting to formal sources across all outcomes collected, ranging from 11 to 25%, however Haiti and Malawi had similar or higher rates of informal disclosure/help-seeking. One explanation for this could be that help-seeking questions were only asked for sexual violence in Tanzania, which typically constitutes a more severe form of violation, more likely to be recognized by both adolescents and adults as abuse. Indeed, in comparison to prevalence among other samples limited to experience of sexual violence, Haiti and Kenya appear similar in terms of informal reporting, while Malawi and Kenya appear comparable in terms of formal disclosure (Appendix I). These patterns reinforce the idea that help-seeking behaviours are likely to vary by type and severity of violence. Additionally, it is possible that national campaigns around HIV or VAC in Tanzania played some role in encouraging disclosure, including implementation of the 2009 Law of the Child Act, in which the Government committed to reforming and strengthening the child protection system, including a structured case management system at multiple levels [28]. However, it is unlikely that the implementation would have resulted in such immediate gains, as the VAC survey data was collected in the same year. In contrast, Cambodia consistently has the lowest prevalence for the two indicators collected (informal and formal disclosure). This may be due to, among others, the higher proportion of the sample living in rural areas, the high levels of self-blame and apathy reported as barriers, and the strong culture of privacy and resistance to disclosing sensitive matters to strangers in the Southeast Asian context [29].

Several patterns emerge that are in line with existing evidence on children’s help-seeking behaviours. Males were less likely to disclose or seek help after experiencing physical and/or sexual violence (including in Haiti, Kenya and Tanzania), although, they were more likely to know where to seek formal help than females (including in Malawi and Nigeria). These results are aligned with Meinck and colleagues’ findings from South Africa [16]. Gender roles and norms may not only promote a social tolerance of violence, but also reinforce negative stereotypes and stigma that lead to a lack of identification and reporting of violence by females, and/or acceptance by males to acknowledge victimization or its impact on their lives [30–35]. Older children and children currently enrolled in school were generally more likely to disclose informally or formally and seek help, perhaps due to increased mobility and financial freedom to access services, as well as potentially larger social networks, leading to increased awareness of what constitutes violence (including in Kenya, Nigeria, and Tanzania) and where to turn for help. This relationship was particularly strong and of high magnitude in Tanzania, which had the lowest comparative rates of school enrolment in the sample (51%). Overall, these results indicate a potential for school-based interventions to play a role in both prevention as well as response to violence [36]. For example, a behaviour change communication toolkit (the ‘Good School Toolkit’) aimed at reducing violence by school staff in primary schools was found to decrease past week physical violence in Uganda [37]. In addition, a short-term classroom-based empowerment and self-defence training was shown to reduce sexual violence and assault among adolescent girls in Malawi and Kenya [38, 39]. At the same time, these results underscore the need to increase efforts to reach more marginalized children who are not in school and may not be able to access school-based services and help.

Mixed findings were found in relation to help-seeking outcomes and correlates with household demographics. This is likely to be, on one hand, due both to the propensity of household members to perpetrate violence, and on the other hand, due to the increased number of individuals in close proximity to whom a child could disclose violence. One hypothesis is that many of the countries analysed have diverse, complex household structures, with protective and risk factors varying by structure. In Nigeria and Tanzania, living without a biological father was correlated with increased formal help-seeking. It is therefore possible that children are more likely to seek formal help when a perpetrator is outside the household, and thus the child is not dependent upon the perpetrator for basic needs and other types of support, leading to a reluctance to report. Indeed, fathers are a key perpetrator of physical violence as shown in the VAC reports [24, 26]. Alternatively, gender differences in prevalence of both experiences of violence and help-seeking suggest that social norms play a large role in determining both, and these may play out differently in households where children live without their biological fathers (either due to an increased likelihood of female headship or multigenerational households headed by a different male relative), leading to differences in attitudes, expectations and ultimately help-seeking behaviours. Relatedly, an increasing number of adult females in the household was associated with increased reporting/help-seeking outcomes in multiple countries, and explanations for this may be similar. At the same time, children in female-headed households were less likely to seek or receive help in two countries, Kenya and Tanzania. In these households, which tend to have a higher likelihood of being in poverty as compared to the general population, limited resources (both financial as well as time, as children may be pulled into productive activities and domestic chores at higher rates), may outweigh the facilitating influence of more progressive gender norms in terms of help-seeking.

Taken together, our findings highlight the importance of contextual factors, including gendered norms around acceptability of VAC and availability of services. They also clearly show that it is not possible to predict who will seek services based on commonly collected program targeting information, including socio-economic status, demographics or urbanicity. For example, one might assume that children in wealthier households in urban areas would be more likely to formally disclose or receive help, however this hypothesis is not consistently supported by the results. Indeed, children’s self-reported reasons for not seeking help—with ‘lack of access’ and ‘financial constraints’ rarely being mentioned as limiting factors. The lack of association between help-seeking and urban status is also surprising, as urban settings tend to have more violence response services available. However, services may still be too limited in terms of population coverage to have an effect, or stigma, norms and attitudes may be a stronger determinant of help-seeking behaviours than availability of services. Thus, before help-seeking can increase, interventions may first be needed to change violence- and gender-related norms.

The limitations of our analysis warrant discussion. First, help-seeking prevalence estimates are based only on those who disclosed their experience of violence and consequent help-seeking behaviours in household surveys, capturing dynamics among a select sample [29]. Likely, the individuals who do not report violence due to stigma, shame or fear of repercussions are also those who might be less likely to have sought help. In this sense, we believe our analysis has captured an upper bound of help-seeking behaviours among children. Secondly, as VACS data is cross-sectional and we do not identify causal links, we can only make claims about associational or correlational relationships. Third, as previously mentioned, country-level indicators and prevalence levels across outcomes are not strictly comparable due to differences in wording and local adaptation of surveys. We assume these adaptations strengthened the ability to capture locally appropriate conceptualization of violence, however recognize that more can be done to tailor international surveys for national application [29]. Nevertheless, there is a tension between using indicators from a standardized core questionnaire to aid in cross-country comparisons versus local adaptation for maximum applicability. Further, sample sizes did not allow sex-disaggregated analyses or for disaggregation by perpetrator, type of violence or poly-victimization experienced due to lack of power. We recognize that help-seeking behaviours and receipt of services is likely to vary by these factors, particularly how normative different types of violence are in a particular setting. This important area for future research may require purposeful samples designed with adequate power to allow multiple comparisons. In addition, we cannot account for help-seeking behaviours among children aged under 13 years, or the frequency or severity of abuse due to data limitations. We are also limited in the data available, including the range of countries with publicly available data, the timing of data collection, and that several countries represent data which are dated. Finally, the data lack measures of available services and other substantive community-level indicators, beyond basic factors such as urban/rural stratification, and living in a camp in Haiti that we were able to include in our analysis.

Improved research methodologies are needed to overcome difficulties with accurately estimating prevalence of sensitive topics such as violence through interview-based surveys. To date, few large-scale surveys in LMICs have utilized self-administered questionnaires to increase reporting, particularly among children [40]. For example, a study in Uganda found that primary school students were seven times more likely to disclose their experience of forced sex, using a sealed-envelope method, compared to face-to-face interviews [41]. Another study of violence among conflict-affected adolescent girls in the Democratic Republic of the Congo and Ethiopia showed that results from participatory group discussions focused on unsafe public spaces, and perpetration by strangers or community members, likely aligned with community norms around “acceptable” violence. However, quantitative results from audio computer-assisted self-interviewing revealed that most violence was perpetrated by boyfriends, husbands or caregivers, thereby highlighting that interview methods and perceived confidentiality may strongly affect responses [40]. Better household and community/environmental-level indicators are needed to understand underlying dynamics, particularly related to parental and guardian characteristics (including mental health, social support, time use, parenting practices), poverty and inequality (ethnicity, disability, labour force and consumption indicators) and service availability [42]. In addition, qualitative work is needed to unpack dynamics around not seeking help among diverse sub-populations to better craft response strategies.

Our results feed into understanding of program and policy options for child survivors of violence, particularly of how to encourage and initiate use of support services for children experiencing violence, and conditions needed before they will do so. One strategy to improve use of services is to address context-specific barriers, including social norms (related to violence and gender), as well as improve integration, linkages and raise awareness of child protection services within health, education, and other social services [10, 43]. Another strategy is to invest additional resources in better supporting those working with children and their families (e.g., health care providers, teachers) to recognize and properly act on signs and symptoms of abuse, while not inadvertently undermining or delegitimizing informal support systems while doing so [44]. Finally, there are likely marginalized populations (e.g., those out of school) where different avenues are needed to reach these children. Analyses presented in this study can help governments and other stakeholders understand the level of investment needed to reach child survivors, and to prioritize interventions aimed at perceived barriers. These recommendations are in line with strategies recognized in recent recommendations to highlight and intensify focus on VAC prevention programmes released by the World Health Organization and partners [35]. We hope that better evidence and methodological innovation will contribute to investments with the potential to decrease the prevalence and incidence of VAC as well as long-lasting negative effects experienced by survivors.

## Conclusion

We found that among children aged 13–17 years experiencing physical and/or sexual violence, informal reporting to or help-seeking from family, friends and neighbours was much more common than formal sources such as medical facilities, police, social workers, or teachers. The most common reasons for not reporting or seeking help included apathy, not needing or wanting services and self-blame. Our analysis elucidated some common patterns of characteristics associated with help-seeking across countries but also many differences, underscoring the need for tailoring interventions aimed at assisting children experiencing violence based on specific contexts and patterns of violence rates, knowledge, and attitudes within countries. This study further highlights the need for multi-sectoral integrated and coordinated approaches for resourcing and expanding use of child protection services within multi-sectoral programming, while combating norms that encourage shame and stigma, and keep violence hidden.

## Data Availability

The data that support the findings of this study are publicly available with permission from host country governments and accessible via the Together for Girls website: https://www.togetherforgirls.org/violence-children-surveys/. We received permission to use these data for the current study.

## Appendix A

**Table 5:** Detailed Information on Survey Implementation, Sampling Procedures, Including Ethical Assurances and Cluster and Split-Sample Design

				Prevalence of lifetime experience of violence by type, and sex
**Country**	**Implementers**	**In-country ethical clearance**	**Ethical considerations**	**% (95% CI)**
Cambodia	National Institute of Statistics (NIS), Ministry of Planning (MoP)	National Ethics Committee for Health Research	Survey introduced as a study to learn about young people’s health, education and life experiences. Supervisors, enumerators and drivers signed confidentiality agreements. All respondents provided with a list of local and national services (incl. Violence response services). Counselling and response services offered to respondents affected by violence through NGO partners in collaboration with the Ministry of Social Affairs, Veterans and Youth Rehabilitation.	PV (*N* = 1164): 59.67; 95% CI: (56.07, 63.27) SV (*N* = 1164): 5.95; 95% CI: (4.29, 7.61) PV and/or SV (*N* = 1164): 60.61; 95% CI: (57.06, 64.17)
Haiti	Interuniversity Institute for Research and Development (INURED)	Ministry of Public Health and Population’s National Ethics Committee	Study described in general terms with a broad list of topics (e.g., health, safety, community violence). All respondents provided with a list of local and regional services as well as a national hotline. Counselling and response services offered to respondents affected by violence through PEPFAR, the Ministry of Health and Population and Partners in Health.	PV (*N* = 1394): 69.78; 95% CI: (66.03, 73.52) SV (*N* = 1376): 27.89; 95% CI: (25.01, 30.76) PV and/or SV (*N* = 1394): 75.48; 95% CI: (71.96, 79.00)
Kenya	Kenya National Bureau of Statistics	Kenya Medical Research Institute	Survey introduced as a study focusing on “health, education and life experiences” of children and youth. All respondents provided with a list of local and regional services as well as a national hotline. Counselling and response services offered to respondents affected by violence through an NGO partner.	PV (*N* = 1288): 79.28; 95% CI: (75.13, 83.43) SV (*N* = 1278): 18.31; 95% CI: (14.85, 21.76) PV and/or SV (*N* = 1289): 81.35; 95% CI: (77.41, 85.28)
Malawi	Centre for Social Research of the University of Malawi	Malawian National Commission for Science and Technology Ethical Review Board	Study was described in general terms with a broad list of topics (e.g., health, safety, community violence) related to the health and life experiences of young people. All respondents provided with a list of local and national services. Free services offered to respondents affected through the Centre for Social Research.	PV (*N* = 1070): 75.8; 95% CI: (71.39, 80.20) SV (*N* = 1070): 27.97; 95% CI: (23.35, 32.60) PV and/or SV (*N* = 1070): 79.31; 95% CI: (75.38, 83.25)
Nigeria	National Population Commission of Nigeria (NPopC)	National Health Ethics Research Committee, National Ministry of Health	Study was described in general terms with a broad list of topics (e.g., health, safety, community violence) related to the health and life experiences of young people. All respondents provided with a list of local and national services. A minimum of one female and one male social worker from the State Ministry of Women Affairs in each geo-political zone (≥ 12 social workers) were on call for referral during the entire survey implementation period.	PV (*N* = 1847): 61.29; 95% CI: (57.66, 64.91) SV (*N* = 1830): 20.22; 95% CI: (17.34, 23.10) PV and/or SV (*N* = 1847): 66.56; 95% CI: (63.10, 70.02)
Tanzania	Muhimbili University of Health and Allied Sciences	Muhimbili University of Health and Allied Sciences Institutional Review Board; Zanzibar Ministry of Health and Social Welfare Institutional Review Board	Study was described in general terms with a broad list of topics (e.g., health, safety, community violence) related to the health and life experiences of young people. All respondents provided with a list of local and regional services. In Mainland Tanzania, the study coordinator worked to find local counselling services. When none were available, he/she deployed a counsellor from Dar es Salaam who provided counselling and made an effort to link the victim with local services. Social welfare officers were contacted in advance to ensure their cooperation if required. In Zanzibar, the study coordinator worked with government district welfare officers to provide counselling and link victims to services.	PV (*N* = 1809): 80.95; 95% CI: (76.24, 85.66) SV (*N* = 1814): 23.14; 95% CI: (19.54, 26.74) PV and/or SV (*N* = 1814): 83.18; 95% CI: (78.99, 87.38)

## Appendix B

**Table 6:** Questions Used in the Compilation of Violence Indicators by Type of Violence and Country

Type of Violence	Country			
	Cambodia	Haiti	Kenya	Malawi
Physical: IPV	Has a romantic partner ever: Slapped or pushed you? Punched, kicked, whipped or beat you with an object? Choked, smothered, tried to drown or burn you intentionally? Used or threatened you with a knife or other weapon?	Not asked	Has your current or previous partner/husband ever: Slapped or pushed you? Hit you with a fist, kicked you, or beat you with an object? Used or threatened to use a knife or other weapon against you?	Have any of your current or previous partners/husband (never, once, a few times, many times): Slapped or pushed you? Punched, kicked, whipped, or beat you with an object? Choked, smothered, tried to drown you, or burned or scalded you intentionally? Used or threatened to use a knife or other weapon against you?
Physical: Peer	Not asked	Not asked	Not asked	Has a person your own age ever: Punched, kicked, whipped or beat you with an object? Choked, smothered, tried to drown you, or burned you intentionally? Used or threatened you with a knife, gun or other weapon?
Physical: Parents/Adults	Has a parent or other adult relative ever: Punched, kicked, whipped or beat you with an object? Choked, smothered, tried to drown or burn you intentionally? Used or threatened you with a knife or other weapon?	Has/did a parent, caregiver, any adult relative, or another adult household member ever: Punch you, kick you, whip you, or beat you with an object? Choke you, smother you or try to drown you? Burn or scald you intentionally (including putting hot pepper in your mouth or on another body part)? Use or threaten to use a knife or other weapon against you?	Has a parent or any adult relative ever: Punched you, kicked you, whipped you, or beat you with an object? Used or threatened to use a knife or other weapon against you?	Has a parent or other relative: Punched, kicked, whipped or beat you with an object? Choked, smothered, or tried to drown, burned or scalded you intentionally? Used or threatened to use a knife or other weapon against you?
Physical: Community	Has one of these [other people in your community] ever: Punched, kicked, whipped or beat you with an object? Choked, smothered, tried to drown or burn you intentionally? Used or threatened you with a knife or other weapon?	Has/did a public authority figure ever: Punch you, kick you, whip you, or beat you with an object? Choke you, smother you or tried to drown you? Burn or scald you intentionally (including putting hot pepper in your mouth or on another body part)? Use or threaten to use a knife or other weapon against you?	Has an authority figure ever: Punched you, kicked you, whipped you, or beat you with an object? Used or threatened to use a knife or other weapon against you?	Has any non-relative community member ever: Punched, kicked, whipped or beat you with an object? Choked, smothered, or tried to drown, burned or scalded you intentionally? Used or threatened to use a knife or other weapon against you?
Sexual: Exploitation	Has anyone ever given you money, food, gifts or any favours to have sexual intercourse or perform any sexual acts with them?	Has anyone ever given you money to have sex with them? Has anyone ever given you food, gifts or any favours so that you have sex with them?	Has anyone ever given you money to have sex with them? Has anybody ever given you food, gifts, or any favours so that you have sex with them?	Has anyone ever given you money, food, gifts, or any favours to have sexual intercourse or perform any other sexual acts with them?
Sexual: Non-contact	Has anyone ever: Made you upset by speaking to you in a sexual way or writing sexual things about you? Forced you to watch sex photos or sex videos against your will? Forced you to be in a sex photo or video against your will?	Not asked	Not asked	Has anyone ever made you upset by speaking to you in a sexual way or writing sexual things about you? Has anyone made you witness sexual activities or sexual abuse, even without making you participate (e.g. images/photos, videos, online) Has anyone made you look at their sexual body parts or made you show them yours?
Sexual: Touching	Has anyone, male or female, ever touched you in a sexual way without your permission, but did not try and force you to have sex of any kind?	How many times in your life has anyone touched you in a sexual way without your permission, but did not try and force you to have sex?	How many times in your life has anyone touched you in a sexual way without your consent, but did not try and force you to have sex?	How many times in your life has anyone touched you in a sexual way without your permission, but did not try and force you to have sex of any kind?
Sexual: Attempted sex	Has anyone ever tried to make you have sexual intercourse of any kind without your permission, but did not succeed?	How many times in your life has anyone tried to make you have sex without your permission, but did not succeed?	How many times in your life has anyone tried to make youhave sex against your will, but did not succeed?	How many times in your life has anyone tried to make you have sexual intercourse of any kind without your permission, but did not succeed?
Sexual: Physically forced sex	Has anyone ever physically forced you to have sexual intercourse of any kind regardless of whether you did or did not fight back?	How many times in your life have you been physically forced to have sex regardless of whether you did or did not fight back?	How many times in your life have you been physically forced to have sex against your will and sexual intercourse was completed?	How many times in your life have you been physically forced to have sexual intercourse of any kind regardless of whether you did or did not fight back?
Sexual: Pressured sex	Has anyone ever pressured you in a non-physical way, to have sexual intercourse of any kind when you did not want to and sex happened?	Have you ever had sex with anyone, male or female, after they pressured you by doing things like telling you lies, making promises about the future they knew were untrue, threatening to end your relationship, or threatening to spread rumours about you? Have you ever had sex with anyone, male or female, after they pressured you by repeatedly asking for sex, or showing they were unhappy? Have you ever had sex with anyone, male or female, after they pressured you using their influence or authority over you, for example, saying they will give you bad grades, that they will fire you or that they will arrest you?	How many times in your life has someone pressured you tohave sex when you did not want to, and sex happened?	How many times in your life has someone pressured you in a nonphysical way, to have sexual intercourse of any kind when you did not want to and sex happened? Have you ever had sexual intercourse of any kind with anyone, male or female, after they pressured you by doing things like telling you lies, making promises about the future they knew were untrue, threatening to end your relationship, or threatening to spread rumours about you? Have you ever had unwanted sexual intercourse of any kind with anyone, male or female, after they pressured you by repeatedly asking for sex, or showing they were unhappy? Have you ever had unwanted sexual intercourse of any kind with anyone, male or female, after they pressured you using their influence or authority over you, for example, saying they will give you bad grades, that they will fire you, or that they will arrest you?
Sexual intercourse: First time	The first time you had sexual intercourse, was it because you wanted to or because you were made to have it without your permission?	The first time you had vaginal or anal intercourse, would you say that you had it because you wanted to, or because you were made to have it against your will?	This first time you had sex, was this something you wanted to do or were you pressured, lured, tricked, physically forced, or threatened in any way?	The first time you had sexual intercourse, would you say that you had it because you wanted to, or because you were made to have it without your permission?
Combined physical violence measure	IPV, parents/adults, community members	Parents/adults, community members	IPV, parents/adults, community members	IPV, parents/adults, community members
Combined sexual violence measure	Touching, attempted sex, forced or pressured sex	Touching, attempted sex, forced or pressured sex	Touching, attempted sex, forced or pressured sex	Touching, attempted sex, forced or pressured sex

Abbreviations: *IPV* Intimate Partner Violence

Note: ^a^All questions taken from questionnaires for females in each country; wording varies slightly for male questionnaires

## Appendix C

**Table 7:** Questions Used in the Compilation of Help-Seeking Indicators for Experience of Physical Violence by Country

	Physical Violence			
	Disclosure	Knowledge of where to seek help	Sought help	Received help
Cambodia	Have you ever told anyone about these experiences? Who did you speak to regarding any physical violence experiences that happened? Own family; husband’s/partner’s family; Current/former husband/partner; current/former boyfriend; friend; neighbour; religious leader; doctor/medical personnel; police; lawyer; social service organization; other; don’t know/declined	Not asked	Thinking about all your experiences with physical violence, have you ever sought help for any of these experiences? From whom have you sought help? Own family; husband’s/partner’s family; Current/former husband/partner; current/former boyfriend; friend; neighbour; religious leader; doctor/medical personnel; police; lawyer; social service organization; other; don’t know/declined	Not asked
Haiti	Did you ever talk to or receive services from a doctor, nurse, or other professional health care worker after any of these experiences when a parent or authority figure was violent towards you? Did you ever talk to or receive services from a lawyer, judge, or anyone else working for an organization other than the police in order to help you have your case reviewed in court after any of these experiences when a parent or authority figure was violent towards you? Did you ever talk to the PNH, BPM, MINUSTAH, UNPOL, security, or protection services after any of these experiences when a parent or authority figure was violent towards you?	Not asked	Not asked	Did you ever receive counselling from a professional after any of these experiences when a parent or authority figure was violent towards you?
Kenya	Not asked	Did you know of a place to go and seek help for any of these violent incidents?	Did you try to seek professional help for any of these incidents?	Were you successful in receiving any professional help for any of these incidents, like from a health facility or NGO?
Malawi	Did you tell anyone about any these experiences? Who did you tell? Mother; father; sister; brother; other relative; husband; boyfriend/romantic partner; friend; neighbour; traditional healer; NGO worker; teacher; employer; community leader; religious leader; other; don’t know/declined	Did you know a hospital/clinic, police station, helpline, social welfare or legal office to go for help?	Did you try to seek help from any of these places for any of these experiences?	Did you receive any help for any of these experiences from a hospital/clinic, police station, helpline, social welfare or legal office? Did you receive help from: Doctor, nurse, or other healthcare worker; police or other security personnel; lawyer, judge/magistrate or other legal professional, other than police; helpline (including phone/internet/website)
Nigeria	Did you tell anyone about any of these experiences? Who did you tell? Mother; father; sister; brother; other relative; husband; boyfriend/romantic partner; friend; neighbour; traditional healer; NGO worker; teacher; employer; neighbourhood leader; religious leader; other	Did you know a hospital/clinic, police station, social worker, teacher, child protection network, NGOs or FBOs to go for help?	Did you try to seek help from any of these places for any of these experiences?	Did you receive any help for any of these experiences from a hospital/clinic, police station, social worker, teacher, child protection network, NGO or FBO? (Yes; no; don’t know/declined) Did you receive help from: A doctor, nurse or other healthcare worker; police or other security personnel; social worker or counsellor; teacher; child protection worker; NGO; FBO

Abbreviations: *BPM* Brigade for the Protection of Minors; *FBO* faith-based organization; *MINUSTAH* La Mission des Nations Unies pour la stabilisation en Haïti; *NGO* non-governmental organization; *PNH* Police Nationale d’Haiti; *UNPOL* United Nations Police

Notes: ^a^Data comes from a nationally representative sample of children aged 13–17 years from Violence Against Children Surveys

^b^Questions are taken from questionnaires used for female respondents

^c^Questions on reasons for not disclosing experiences of violence or not seeking help, extended help seeking during most recent episode of violence experience, and additional services respondent would have liked, are not included in this table

^d^Tanzania did not ask questions on physical violence help seeking behaviour

## Appendix D

**Table 8:** Questions Used in the Compilation of Help-Seeking Indicators for Experience of Sexual Violence by Country

	Sexual Violence			
	Disclosure	Knowledge of where to seek help	Sought help	Received help
Cambodia	Have you ever told anyone about these experiences? Who did you speak to regarding any of these sexual experiences that happened without your permission?Own family; husband’s/partner’s family; Current/former husband/partner; current/former boyfriend; friend; neighbour; religious leader; doctor/medical personnel; police; lawyer; social service organization; other; don’t know/declined	Not asked	Thinking about all of the sexual experiences that happened without your permission, have you ever sought help for these experiences? From whom have you sought help? Own family; husband’s/partner’s family; Current/former husband/partner; current/former boyfriend; friend; neighbour; religious leader; doctor/medical personnel; police; lawyer; social service organization; other; don’t know/declined	Not asked
Haiti	Did you ever tell anybody about any of these experiences- unwanted touching, attempted sex, pressured sex, or physically forced sex? Who were the people you spoke to? Mother; father; sister; brother; other relative; husband; boyfriend/romantic partner; friend; neighbour; doctor/health care provider; counsellor; traditional healer; hotline; NGO worker; teacher; employer; police; Minustah/UNPOL; other security person; community leader; religious leader; lawyer or legal aid; other; don’t know/declined Did you ever talk to or receive services from a doctor, nurse, or other professional health care worker after any of your experiences of sexual violence that we have talked about/discussed? Did you ever talk to or receive services from a lawyer, judge, or anyone else working for an organization other than the police in order to help you have your case reviewed in court? Did you ever talk to the PNH, BPM, MINUSTAH, UNPOL, security, or protection services?	Not asked	Not asked	Did you ever receive counselling from a professional?
Kenya	Did you ever tell anybody about any of these incidents unwanted touching, attempted sex, physically forced sex, or pressured sex? Was a relative among the people you spoke to? What was their reaction? Who were the relatives you spoke to? Father; mother; brother; sister; uncle; aunt; other male relative; other female relative; don’t know; refused Was a boyfriend, romantic partner, or husband among the people you spoke to? Was an authority figure, such as a teacher or police, among the people you spoke to? Was a friend among the people you spoke to? Was there anyone else you spoke to?	Did you know of a place to go and seek professional help for any of these sexual incidents?	Did you try to seek professional help for any of these incidents?	Were you successful in receiving any professional help for any of these incidents, like from a clinic or NGO?
Malawi	Did you tell anyone about any these experiences? Who did you tell? Mother; father; sister; brother; other relative; husband; boyfriend/romantic partner; friend; neighbour; traditional healer; NGO worker; teacher; employer; community leader; religious leader; other; don’t know/declined	Did you know a hospital/clinic, police station, helpline, social welfare or legal office to go for help?	Did you try to seek help from any of these places for any of these experiences?	Did you receive any help for any of these experiences from a hospital/clinic, police station, helpline, social welfare or legal office? Did you receive help from: Doctor, nurse, or other healthcare worker; police or other security personnel; lawyer, judge/magistrate or other legal professional, other than police; a social worker or counsellor; helpline (including phone/internet/website
Nigeria	Did you tell anyone about any of these experiences? Who did you tell? Mother; father; sister; brother; other relative; husband; boyfriend/romantic partner; friend; neighbour; traditional healer; NGO worker; teacher; employer; neighbourhood leader; religious leader; other	Thinking about all your unwanted sexual experiences, did you know a hospital/clinic, police station, social worker, teacher, child protection network, NGOs or FBOs to go for help?	Did you try to seek help from any of these places for any of these experiences?	Did you receive any help for any of these experiences from a hospital/clinic, police station, social worker, teacher, child protection network, NGO or FBO? Did you receive help from: A doctor, nurse or other healthcare worker; police or other security personnel; social worker or counsellor; teacher; child protection worker; NGO; FBO
Tanzania	Now I would like you to think back to all encounters concerning sexual contacts against your will, unsuccessful sexual attempts and forced sexual intercourse incidents which you have just told me about. Did you ever tell anybody about these incidents? Whom did you tell about these happenings? Mother; father; husband/my lover; another brother who is male; friend; teacher; religious leader; another sister who is female; health worker (doctor or nurse); traditional witchdoctor; advice; elder/community leader; police; another person; does not know; does not want to answer	Not asked	Did you try to seek help for any of these incidents?	Did you succeed to get professional assistance or any assistance in any of these incidents?

Abbreviations: *BPM* Brigade for the Protection of Minors; *FBO* faith-based organization; *MINUSTAH* La Mission des Nations Unies pour la stabilisation en Haïti; *NGO* non-governmental organization; *PNH* Police Nationale d’Haiti; *UNPOL* United Nations Police

Notes: ^a^Data comes from a nationally representative sample of children aged 13–17 years from Violence Against Children Surveys

^b^Questions are taken from questionnaires used for female respondents

^c^Questions on reasons for not disclosing experiences of violence or not seeking help, extended help seeking during most recent episode of violence experience, and additional services respondent would have liked, are not included in this table

## Appendix E

**Table 9:** Descriptive Statistics for Background Characteristics From Children Experiencing Physical and/or Sexual Violence in the Violence Against Children Surveys by Country

	Cambodia		Haiti	
Variable	% or prevalence	95% CI	% or prevalence	95% CI
Male	54.19	(45.69, 62.70)	51.14	(43.85, 58.44)
Age in years	14.80	(14.67, 14.93)	14.96	(14.85, 15.07)
Currently enrolled in school	70.08	(65.03, 75.13)	86.31	(83.62, 89.00)
Not living with biological mother	11.98	(8.72, 15.24)	30.78	(26.87, 34.70)
Not living with biological father	19.02	(14.84, 23.20)	44.91	(40.87, 48.95)
Number of children 0–17 years in household	2.62	(2.50, 2.73)	3.49	(3.33, 3.65)
Number of males 18+ in household	1.49	(1.39, 1.59)	1.57	(1.47, 1.67)
Number of females 18+ in household	1.61	(1.53, 1.70)	1.67	(1.58, 1.76)
Female head of household	17.13	(13.59, 20.66)	52.45	(46.97, 57.93)
Poorer wealth quintile	24.01	(19.57, 28.45)	17.90	(14.15, 21.64)
Middle wealth quintile	21.14	(17.25, 25.04)	18.59	(15.08, 22.09)
Richer wealth quintile	21.48	(16.91, 26.04)	19.98	(15.41, 24.55)
Richest wealth quintile	14.49	(9.32, 19.65)	24.90	(19.78, 30.02)
Urban	15.55	(9.57, 21.53)	41.13	(32.58, 49.67)
Camp			1.59	(0.19, 2.99)
N	586		1040	

Abbreviations: *CI* Confidence Interval; *N* Number of observations

Notes: ^a^Estimates are n (weighted %) or prevalence (95% Confidence Interval)

^b^Data comes from a nationally representative sample of children aged 13–17 years from Violence Against Children Surveys

^c^Samples from male and female respondents are combined due to low overall help seeking rates within subsamples

^d^Wealth quintiles are from indices created through factor analysis of household assets and dwelling characteristics, similar to standard methodology from the Demographic and Health Surveys and are age-group specific

## Appendix F

**Table 10:** Descriptive Statistics for Background Characteristics From Children Experiencing Physical and/or Sexual Violence in the Violence Against Children Surveys by Country

	Kenya		Malawi	
Variable	% or prevalence	95% CI	% or prevalence	95% CI
Male	51.95	(42.55, 61.35)	53.91	(41.96, 65.85)
Age in years	14.93	(14.80, 15.06)	14.75	(14.63, 14.88)
Currently enrolled in school	84.47	(81.18, 87.76)	85.17	(81.00, 89.33)
Not living with biological mother	21.13	(17.00, 25.26)	28.95	(24.87, 33.04)
Not living with biological father	42.29	(37.69, 46.89)	49.24	(44.32, 54.17)
Number of children 0–17 years in household	2.44	(2.27, 2.60)	3.52	(3.34, 3.71)
Number of males 18+ in household (adults in Kenya)	0.81	(0.69, 0.94)	1.22	(1.12, 1.32)
Number of females 18+ in household			1.37	(1.27, 1.46)
Female head of household	41.93	(37.68, 46.18)	43.16	(37.40, 48.93)
Poorer wealth quintile	19.63	(15.88, 23.39)	20.12	(16.35, 23.89)
Middle wealth quintile	19.80	(15.98, 23.63)	24.60	(19.67, 29.53)
Richer wealth quintile	19.42	(15.78, 23.05)	21.82	(17.32, 26.32)
Richest wealth quintile	18.67	(14.43, 22.90)	17.39	(12.28, 22.51)
Urban	17.78	(10.68, 24.89)	16.30	(9.50, 23.09)
N	1005		864	

Abbreviations: *CI* Confidence Interval; *N* Number of observations

Notes: ^a^Estimates are n (weighted %) or prevalence (95% Confidence Interval)

^b^Data comes from a nationally representative sample of children aged 13–17 years from Violence Against Children Surveys

^c^Samples from male and female respondents are combined due to low overall help seeking rates within subsamples

^d^Wealth quintiles are from indices created through factor analysis of household assets and dwelling characteristics, similar to standard methodology from the Demographic and Health Surveys and are age-group specific

^e^In Kenya, the number of adults and children in household refer to the number of adults and children in the same sleeping areas as the respondent

## Appendix G

**Table 11:** Descriptive Statistics for Background Characteristics From Children Experiencing Physical and/or Sexual Violence in the Violence Against Children Surveys by Country

	Nigeria		Tanzania	
Variable	% or prevalence	95% CI	% or prevalence	95% CI
Male	51.02	(44.25, 57.79)	32.36	(18.42, 46.31)
Age in years	14.83	(14.72, 14.94)	15.56	(15.25, 15.88)
Currently enrolled in school	73.11	(68.92, 77.30)	51.46	(40.32, 62.60)
Not living with biological mother	19.99	(16.88, 23.11)	38.16	(29.51, 46.82)
Not living with biological father	26.07	(22.82, 29.33)	48.75	(38.75, 58.75)
Number of children 0–17 years in household	3.44	(3.22, 3.65)	3.99	(3.61, 4.37)
Number of males 18+ in household	1.59	(1.48, 1.71)	0.83	(0.64, 1.02)
Number of females 18+ in household	1.67	(1.56, 1.78)	1.08	(0.89, 1.26)
Female head of household	18.50	(15.65, 21.35)	54.98	(43.54, 66.42)
Poorer wealth quintile	18.68	(15.73, 21.63)	19.25	(10.89, 27.61)
Middle wealth quintile	19.93	(16.90, 22.96)	14.56	(7.80, 21.33)
Richer wealth quintile	22.68	(19.28, 26.08)	17.34	(7.42, 27.25)
Richest wealth quintile	22.63	(18.26, 27.00)	23.58	(10.54, 36.62)
Urban	39.89	(33.34, 46.45)		
Zanzibar			1.05	(0.64, 1.47)
N	1185		219	

Abbreviations: *CI* Confidence Interval; *N* Number of observations

Notes: ^a^Estimates are n (weighted %) or prevalence (95% Confidence Interval)

^b^Data comes from a nationally representative sample of children aged 13–17 years from Violence Against Children Surveys

^c^Samples from male and female respondents are combined due to low overall help seeking rates within subsamples

^d^Wealth quintiles are from indices created through factor analysis of household assets and dwelling characteristics, similar to standard methodology from the Demographic and Health Surveys and are age-group specific

## Appendix H

**Table 12:** Descriptive Statistics for Help-Seeking Behaviours from Children Experiencing Violence by Type of Violence and Country

		Physical violence				
Country		Informal: Disclosure	Formal: Knowledge of where to seek help	Formal: Disclosure	Formal: Help seeking	Formal: Received help
Cambodia	Prevalence	23.13		0.11	0.00	
	(95% CI)	(19.26, 27.00)		(−0.11, 0.33)	–	
	N	570		570	575	
Haiti	Prevalence			7.25		
	(95% CI)			(4.85, 9.65)		
	N			966		
Kenya	Prevalence		13.19		2.26	1.44
	(95% CI)		(10.12, 16.27)		(1.21, 3.31)	(0.57, 2.32)
	N		971		971	971
Malawi	Prevalence	48.69	2.42	24.94	9.53	7.25
	(95% CI)	(44.69, 52.69)	(0.67, 4.17)	(19.51, 30.37)	(5.33, 13.74)	(3.04, 11.45)
	N	823	823	821	821	821
Nigeria	Prevalence	38.43	18.34	1.66	1.65	1.06
	(95% CI)	(34.70, 42.16)	(14.92, 21.76)	(0.71, 2.62)	(0.75, 2.55)	(0.27, 1.84)
	N	1095	1103	1095	1105	1105

Abbreviations: *CI* Confidence Interval; *N* Number of observations

Notes: ^a^Estimates are n (weighted %) or prevalence (95% Confidence Interval)

^b^Data comes from a nationally representative sample of children aged 13–17 years from Violence Against Children Surveys

^c^Cambodia did not ask questions on knowledge of where to seek formal help or on receiving formal help

^d^In Haiti, informal disclosure was asked for experience of sexual violence only. Due to the wording of questions, we were unable to parse out formal disclosure from receiving help for experience of physical violence. Haiti did not ask questions on knowledge of where to seek help

^e^In Kenya, disclosure, both formal and informal, was asked for experience of sexual violence only

^f^In Tanzania, help seeking questions were asked for experience of sexual violence only and are therefore not included in this table (as they are listed in Table 2). Questions on knowledge of where to seek formal help were not asked

## Appendix I

**Table 13:** Descriptive Statistics for Help-Seeking Behaviours from Children Experiencing Violence by Type of Violence and Country

		Sexual violence				
Country		Informal: Disclosure	Formal: Knowledge of where to seek help	Formal: Disclosure	Formal: Help seeking	Formal: Received help
Cambodia	Prevalence	16.57		0.00	0.00	
	(95% CI)	(1.90, 31.24)		–	–	
	N	52		52	57	
Haiti	Prevalence	42.40		0.95	6.44	
	(95% CI)	(35.37, 49.42)		(−0.36, 2.25)	(3.40, 9.48)	
	N	385		385	385	
Kenya	Prevalence	31.67	8.27	22.84	4.45	3.12
	(95% CI)	(21.70, 41.64)	(−1.18, 17.72)	(14.63, 31.06)	(0.79, 8.11)	(0.53, 5.70)
	N	191	191	186	186	186
Malawi	Prevalence	43.26	1.18	25.10	6.43	3.63
	(95% CI)	(33.99, 52.52)	(−0.31, 2.66)	(17.74, 32.46)	(2.27, 10.58)	(0.25, 7.01)
	N	270	270	268	268	268
Nigeria	Prevalence	32.65	14.50	1.55	1.98	1.57
	(95% CI)	(25.95, 39.36)	(9.91, 19.09)	(0.19, 2.90)	(0.35, 3.61)	(0.16, 2.99)
	N	339	339	343	348	348

Abbreviations: *CI* Confidence Interval; *N* Number of observations

Notes: ^a^Estimates are n (weighted %) or prevalence (95% Confidence Interval)

^b^Data comes from a nationally representative sample of children aged 13–17 years from Violence Against Children Surveys

^c^Cambodia did not ask questions on knowledge of where to seek formal help or on receiving formal help

^d^In Haiti, informal disclosure was asked for experience of sexual violence only. Due to the wording of questions, we were unable to parse out formal disclosure from receiving help for experience of physical violence. Haiti did not ask questions on knowledge of where to seek help

^e^In Kenya, disclosure, both formal and informal, was asked for experience of sexual violence only

^f^In Tanzania, help seeking questions were asked for experience of sexual violence only and are therefore not included in this table (as they are listed in Table 2). Questions on knowledge of where to seek formal help were not asked

## Appendix J

**Table 14:** Reported Main Reason for not Seeking Help Among Children Aged 13–17 Years Who Ever Experienced Physical and/or Sexual Violence by Country

	Cambodia		Kenya		Malawi		Nigeria	
	Physical violence	Sexual violence	Physical violence	Sexual violence	Physical violence	Sexual violence	Physical violence	Sexual violence
	%	%	%	%	%	%	%	%
Lack of awareness	7.65	2.13	na	na	na	na	na	na
Lack of access	0.36	na	5.69	0.00	na	na	na	na
Afraid of repercussions	5.69	8.51	26.83	11.11	5.04	10.20	10.00	10.00
Shame and stigma	11.74	6.38	4.07	19.44	0.72	10.20	1.11	6.00
Self-blame	55.69	0.00	na	na	17.99	6.12	14.44	2.00
Financial constraints	0.00	na	0.00	2.78	1.44	2.04	1.11	4.00
Lack of social support	2.31	2.13	1.63	2.78	2.88	0.00	7.78	0.00
Apathy	8.54	55.32	37.40	41.67	34.53	38.78	38.89	50.00
Perceived helplessness	3.91	na	na	na	na	na	na	na
Did not need or want services	1.25	14.89	13.01	11.11	12.95	14.29	21.67	24.00
Other	2.85	10.64	11.38	11.11	24.46	18.37	5.00	4.00
N	562	47	123	36	139	49	180	50

Abbreviations: *CI* Confidence Interval; *na* not asked; *N* Number of observations

Notes: ^a^Estimates are %

^b^Data comes from a nationally representative sample of children aged 13–17 years from Violence Against Children Surveys. Samples from male and female respondents are combined due to low overall help seeking rates within subsamples. These questions were asked only for those who had ever experienced physical or sexual violence.

^c^Questions were categorized as follows: lack of awareness (did not know where to go); lack of access (too far to services); afraid of repercussions (afraid of causing more violence or getting in trouble, did not want abuser to get in trouble, perpetrator threatened me, was or felt threatened); shame and stigma (afraid of being mocked, embarrassed for self or family, felt ashamed); financial constraints (could not afford services, could not afford transport); lack of social support (afraid of being abandoned, dependent on perpetrator, no one to help me); apathy (did not think it was a problem); perceived helplessness (felt it was useless); did not need or want services; and other reasons

^d^In Kenya, respondents could provide multiple reasons

## Appendix K

**Table 15:** Reported Main Reason for not Seeking Help Among Children Aged 13–17 Years Who Ever Experienced Physical and/or Sexual Violence by Country

	Haiti							
	Physical violence				Sexual violence			
	Health	Law	Police	Counseling	Health	Law	Police	Counseling
	%	%	%	%	%	%	%	%
Lack of awareness	7.35	6.63	6.05	15.14	10.16	10.03	7.59	21.33
Lack of access	1.30	0.93	0.70	0.84	1.27	0.94	1.27	0.67
Afraid of repercussions	2.61	5.47	5.36	1.92	7.62	9.40	11.71	5.00
Shame and stigma	0.24	0.70	0.47	0.24	2.22	2.19	2.85	0.67
Financial constraints	4.27	1.40	0.47	1.20	4.76	1.57	0.95	2.33
Lack of social support	2.84	2.56	3.03	4.57	2.86	3.76	4.75	7.00
Apathy	18.48	20.23	18.63	16.59	15.56	17.24	13.92	11.00
Perceived helplessness	21.68	22.91	25.15	20.79	20.00	21.32	21.52	16.33
Did not need or want services	27.96	27.91	31.20	30.65	25.40	21.63	25.00	28.00
Other	13.27	11.28	8.96	8.05	10.16	11.91	10.44	7.67
N	844	860	859	832	315	319	316	300

Abbreviations: *CI* Confidence Interval; *na* not asked; *N* = Number of observations

Notes: ^a^Estimates are %

^b^Data comes from a nationally representative sample of children aged 13–17 years from Violence Against Children Surveys. Samples from male and female respondents are combined due to low overall help seeking rates within subsamples. These questions were asked only for those who had ever experienced physical or sexual violence

^c^Questions were categorized as follows: lack of awareness (did not know where to go); lack of access (too far to services); afraid of repercussions (afraid of causing more violence or getting in trouble, did not want abuser to get in trouble, perpetrator threatened me, was or felt threatened); shame and stigma (afraid of being mocked, embarrassed for self or family, felt ashamed); financial constraints (could not afford services, could not afford transport); lack of social support (afraid of being abandoned, dependent on perpetrator, no one to help me); apathy (did not think it was a problem); perceived helplessness (felt it was useless); did not need or want services; and other reasons

## Abbreviations

CIs: Confidence intervals
IPV: Intimate partner violence
LMICs: Low- and middle-income countries
NGO: Non-governmental organization
OR: Odds Ratio
UNICEF: United Nations Children’s Fund
VAC: Violence against children
VACS: Violence Against Children Surveys
